# Prognostic Value of Inflammatory Hematological Indices for In-Hospital Mortality After Stroke

**DOI:** 10.3390/medicina62030441

**Published:** 2026-02-26

**Authors:** Nazira Zharkinbekova, Gulnur Arykbayeva, Gulnara Mustapayeva, Ainur Yessetova, Murat Suleimenov, Gaukhar Tolebayeva, Aigul Turtayeva, Altynay Yelubayeva, Sandugash Rustemova, Dinara Tileuberdiyeva, Zaure Suleimenova, Aziza Mukasheva

**Affiliations:** 1 Department of Neurology, Psychiatry, Rehabilitology, and Neurosurgery, South Kazakhstan Medical Academy, Shymkent 160019, Kazakhstan; nazirazhar@mail.ru (N.Z.); gul_nara78@mail.ru (G.M.); esetova.aynura@mail.ru (A.Y.); suleymenov_mura@mail.ru (M.S.); tolebaeva79@mail.ru (G.T.);; 2 Department of Therapy and Cardiology, South Kazakhstan Medical Academy, Shymkent 160019, Kazakhstan; 3 Clinic-Diagnostic Centre, Akhmet Yassawi International Kazakh-Turkish University, Turkestan 161200, Kazakhstan; 4 Regional Clinical Hospital, Stroke Center, Shymkent 160019, Kazakhstan; 5 Department of General Medical Practice and Dermatovenereology, South Kazakhstan Medical Academy, Shymkent 160019, Kazakhstan; azizafeat@mail.ru

**Keywords:** stroke, neutrophil-to-lymphocyte ratio, inflammatory indices, neuroinflammation

## Abstract

*Background and Objectives*: This study aimed to assess the prognostic value of several inflammation-based hematological indices in patients with ischemic and hemorrhagic stroke and to evaluate whether their prognostic significance differs between stroke types. The analyzed indices included the neutrophil-to-lymphocyte ratio (NLR), platelet-to-lymphocyte ratio (PLR), lymphocyte-to-monocyte ratio (LMR), systemic inflammatory response index (SIRI), systemic immune-inflammation index (SII), and aggregate index of systemic inflammation (AISI). *Materials and Methods*: This retrospective cohort study analyzed the medical records of stroke patients admitted to two hospitals in Shymkent, Kazakhstan. Hematological parameters were calculated from routine complete blood counts obtained on the third day of hospitalization. Nonparametric tests, univariable and multivariable logistic regression, and receiver operating characteristic (ROC) analysis were used to evaluate associations between inflammatory indices and in-hospital mortality. *Results*: A total of 199 patients who met the inclusion criteria were classified into three groups according to in-hospital outcome at discharge: (1) patients discharged alive (favorable outcome), (2) patients who died during hospitalization due to ischemic stroke (unfavorable ischemic stroke), and (3) patients who died during hospitalization due to hemorrhagic stroke (unfavorable hemorrhagic stroke). NLR, SIRI, AISI, and SII values were significantly higher in both unfavorable outcome groups compared with the favorable outcome group (*p* < 0.001, effect size r > 0.6). No statistically significant differences were observed between unfavorable ischemic and hemorrhagic stroke outcomes. In logistic regression analysis, NLR (OR = 1.65) and SIRI (OR = 2.36) showed the strongest associations with in-hospital mortality. ROC analysis demonstrated good predictive performance, with AUC values of 0.885 for NLR and 0.867 for SIRI. *Conclusions*: The inflammatory indices evaluated in this study were associated with stroke outcomes regardless of stroke subtype. Among them, SIRI and NLR showed the highest prognostic value. These indices may serve as accessible markers of disease severity but should not be considered independent clinical decision-making tools.

## 1. Introduction

Stroke remains one of the leading causes of disability and mortality worldwide. According to the World Stroke Organization (Global Stroke Fact Sheet 2025), stroke is the second leading cause of death and the third leading cause of combined death and disability, measured by disability-adjusted life years [1]. In recent years, increasing attention has been paid to neuroinflammation as a key mechanism in stroke pathogenesis. Ischemic brain injury triggers a complex inflammatory cascade involving sequential activation of circulating blood cells and local immune responses [2,3,4]. Several recent studies have demonstrated that elevated inflammatory indices, such as NLR and SII, are associated with increased stroke severity and worse clinical outcomes [5,6,7]. In the acute phase of ischemia, the inflammatory cascade response involves the rapid and coordinated activation of immune cells. Neutrophils infiltrate injured tissues within minutes to hours, releasing reactive oxygen species, cytokines, and proteolytic enzymes that disrupt the blood–brain barrier (BBB) [8,9]. Lymphocyte suppression and migration of T cell subsets further contribute to immune regulation [10], whereas monocytes differentiate into macrophages with both inflammatory and reparative functions [11]. Platelet interactions amplify vascular injury through thrombo-inflammatory mechanisms [12,13]. As these pathways are reflected in blood parameters such as NLR, SII, SIRI, and AISI, their elevation in patients with stroke may reflect the intensity of systemic inflammatory response during the acute phase of stroke. Therefore, hematological markers associated with inflammation are gaining attention as accessible and cost-effective biomarkers for stroke prognosis. Thus, the aim of this study was to compare the prognostic significance of simple hematological indices (NLR, PLR, LMR, SIRI, SII, and AISI) for in-hospital mortality of stroke and to assess whether their prognostic value differs between ischemic and hemorrhagic strokes.

## 2. Materials and Methods

### 2.1. Study Design and Population

The medical records of 432 patients with ischemic or hemorrhagic stroke were retrospectively analyzed. All patients were admitted to the Stroke Center of the Regional Clinical Hospital and City Clinical Hospital No. 1 in Shymkent, Kazakhstan, between 2023 and 2024.

The inclusion criteria were age ≤ 85 years, a confirmed diagnosis of ischemic or hemorrhagic stroke, hospitalization in the neurology department, and the availability of complete medical records with hematological data obtained from the third day of hospitalization. The exclusion criteria included age > 85 years, a history of transient ischemic attack, recent major surgery, malignancy, chronic inflammatory or severe somatic diseases, early death within the first 1–2 days of hospitalization, and incomplete documentation. Patients were classified into three groups based on stroke type and in-hospital outcome at discharge: (1) favorable outcome, defined as survival to hospital discharge; (2) unfavorable outcome due to ischemic stroke, defined as in-hospital mortality; and (3) unfavorable outcome due to hemorrhagic stroke, defined as in-hospital mortality. The favorable outcome group was used as the reference group for comparative analyses. Given the retrospective design, the analysis was observational in nature, and the results should be interpreted as associations rather than causal relationships.

### 2.2. Laboratory Parameters and Inflammatory Indices

Hematological indices were analyzed using data from complete blood counts expressed as ×10^9^/L and leukocyte differentials obtained on the third day after admission to the neurology department. Complete blood counts were performed using automated hematology analyzers according to standard laboratory protocols. All measurements were conducted in certified hospital laboratories with routine internal quality-control procedures. Based on experimental and clinical evidence indicating that systemic inflammatory responses and leukocyte activation peak approximately 48–72 h after stroke onset, corresponding to a more stable subacute phase of inflammation [14,15,16,17], blood samples were collected on the third day after hospitalization. This time point was selected to minimize the influence of early stress-related hematological fluctuations observed during the first hours after stroke and to better reflect stabilized systemic inflammatory activity. Systemic inflammatory indices were calculated using the following standard formulas: neutrophil-to-lymphocyte ratio (NLR), platelet-to-lymphocyte ratio (PLR), lymphocyte-to-monocyte ratio (LMR), systemic inflammatory response index (SIRI = (neutrophils × monocytes)/lymphocytes), systemic immune-inflammation index (SII = platelets × (neutrophils/lymphocytes)), and aggregate index of systemic inflammation (AISI = (neutrophils × monocytes × platelets)/lymphocytes).

### 2.3. Statistical Analysis

Statistical analysis was performed with Jamovi version 2.3.28 (The Jamovi project, 2021; www.jamovi.org) and Python version 3.13.1 (Python Software Foundation; https://www.python.org; accessed 10 January 2026). Continuous variables showed non-normal distributions; therefore, nonparametric statistical methods were applied. The outcome variable was coded as 0 = favorable and 1 = unfavorable. Differences between the groups were assessed using the Mann–Whitney U test. Binary logistic regression was used to evaluate the association between inflammatory markers and stroke outcomes. Results are presented as odds ratios (ORs) with 95% confidence intervals (Cls). Receiver operating characteristic curve analysis was performed to assess the predictive value of the individual inflammatory indices. Odds ratios in logistic regression models were calculated using continuous variables. ROC-derived cutoff values were not used for regression modeling. To identify independent predictors of in-hospital mortality, a multivariable logistic regression analysis was conducted. In-hospital mortality was used as the dependent variable. Age was included as a continuous variable. Sex was coded as a binary variable (0 = male, 1 = female). Stroke type (ischemic or hemorrhagic) and inflammatory indices (NLR and SIRI) were included as covariates. Statistical significance was set at *p* < 0.05 and high significance at *p* < 0.001.

## 3. Results

### 3.1. Study Population

After applying the inclusion and exclusion criteria, outlier detection was performed using *Z*-score analysis for continuous inflammatory indices. Observations with absolute *Z*-scores > 3 were considered extreme values and excluded from the final dataset. A total of 16 cases (7.4% of the initial sample) were removed. After outlier exclusion, 199 patients were included in the final analysis. The study included 69 patients with a favorable outcome, 72 with an unfavorable ischemic stroke outcome, and 58 with an unfavorable hemorrhagic stroke outcome. Outlier removal was performed to reduce the influence of extreme observations on regression estimates and to improve model stability.

The mean age of the study population was similar across the outcome groups. Patients with a favorable outcome had a mean age of 63.1 ± 10.2 years, while patients with unfavorable ischemic and hemorrhagic stroke had mean ages of 66.8 ± 9.8 and 59.4 ± 12.1 years, respectively. The proportion of men and women was also comparable between the groups. No statistically significant differences in age or sex distribution were observed; therefore, these variables were not included in the univariate group comparisons but were assessed in the multivariable regression analysis.

### 3.2. Comparison of Hematological Indices Between Groups

Based on the Mann–Whitney U test results (Table 1), NLR, AISI, SIRI, and SII values were significantly higher in both unfavorable ischemic stroke and unfavorable hemorrhagic stroke groups compared with the favorable outcome group (all *p* < 0.001), with large effect sizes (r > 0.6). In contrast, PLR and LMR did not show clinically relevant differences between outcome groups. Importantly, no statistically significant differences were observed between unfavorable ischemic and hemorrhagic stroke outcomes for any inflammatory index (all *p* > 0.05; effect sizes r < 0.1). Therefore, patients with unfavorable outcomes were combined into a single group for subsequent analyses.

### 3.3. Logistic Regression Analysis

Univariable logistic regression analysis (Table 2) showed that the most significant predictors of adverse outcomes were NLR and SIRI, with OR values of 1.647 (95% CI: 1.37–1.97, *p* < 0.001) and 2.360 (95% CI: 1.7–3.2, *p* < 0.001), respectively. The confidence intervals for both indices exceeded one, supporting the reliability of the associations. In addition to NLR and SIRI, the AISI index demonstrated moderate predictive value in identifying adverse outcomes. The optimal threshold value for AISI was 530.6, with a sensitivity of 76.1% and specificity of 85.5%. Other indices, such as LMR, PLR, and SII, also reached statistical significance but showed lower prognostic performance.

### 3.4. Multivariable Logistic Regression Analysis

In the multivariable logistic regression analysis (Table 3), age and NLR remained independently associated with in-hospital mortality. Each one-year increase in age was associated with an approximately 8% increase in the odds of death (OR 1.09, 95% CI 1.03–1.15; *p* = 0.003). Higher NLR values were also independently associated with an unfavorable outcome (OR 1.71, 95% CI 1.25–2.34; *p* < 0.001). SIRI showed a positive association with in-hospital mortality; however, this association did not reach statistical significance after adjustment (OR 1.56, 95% CI 0.96–2.52; *p* = 0.073). Although inflammatory indices did not differ between stroke subtypes in stratified analysis, stroke type itself remained independently associated with mortality risk in the multivariable model. Hemorrhagic stroke was associated with a substantially higher risk of in-hospital mortality compared with ischemic stroke (OR 17.9, 95% CI 4.7–68.3; *p* < 0.001). Sex was not independently associated with outcome.

### 3.5. ROC Curve Analysis

**Figure 1:**
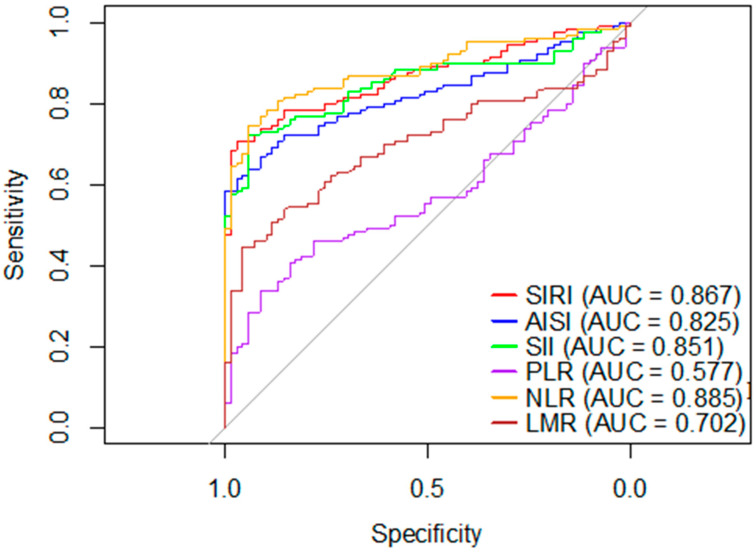
Receiver operating characteristic curves (ROC) of inflammatory and hematological indices for predicting unfavorable in-hospital stroke outcomes. The gray diagonal line represents the line of no discrimination (AUC = 0.5). Abbreviations: ROC, receiver operating characteristic curve; AUC, area under the curve. SIRI, systemic inflammatory response index; AISI, aggregate index of systemic inflammation; SII, systemic immune-inflammation index; PLR, platelet-to-lymphocyte ratio; NLR, neutrophil-to-lymphocyte ratio; LMR, lymphocyte-to-monocyte ratio. NLR and SIRI demonstrated the highest discriminative performance, with AUC values of 0.885 and 0.867, respectively. AISI and SII showed moderate predictive ability, whereas PLR demonstrated limited discriminative performance.

Based on ROC analysis, NLR and SIRI demonstrated the highest discriminatory ability for unfavorable in-hospital outcomes, with AUC values of 0.885 and 0.867, respectively (Figure 1). The cutoff values of 4.8 for NLR and 2.7 for SIRI were identified to describe diagnostic performance, including sensitivity and specificity. These cutoff values were used for classification purposes only and were not applied in logistic regression models for risk estimation. The other inflammatory indices exhibited lower discriminatory performance.

### 3.6. Distributions of Indices Across Study Groups

**Figure 2:**
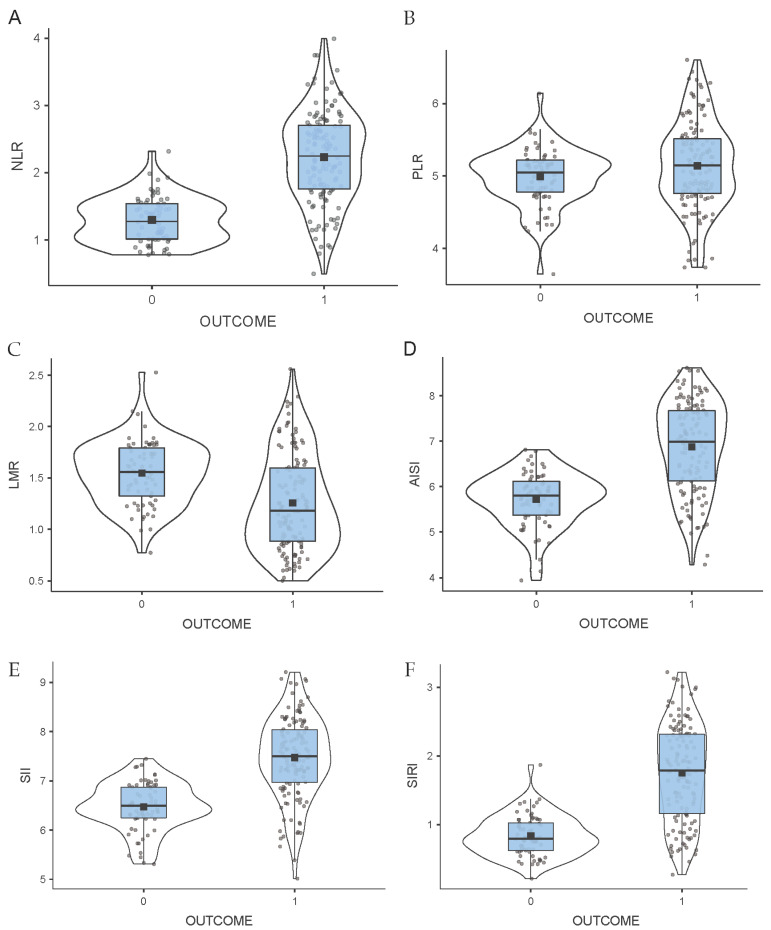
Violin plots of log-transformed inflammatory indices according to clinical outcome (0 = favorable outcome; 1 = unfavorable outcome). Violin plots showing the distribution of inflammation indices according to clinical outcome. The panels represent: (**A**) NLR, (**B**) PLR, (**C**) LMR, (**D**) AISI, (**E**) SII, and (**F**) SIRI, shown as log-transformed variables. The violin shape represents the core of the data density distribution. The embedded box plot shows the interquartile range (IQR), the horizontal line inside the box represents the median, and the square marker indicates the mean. Individual data points are shown as dots. Abbreviations: NLR, neutrophil-to-lymphocyte ratio; PLR, platelet-to-lymphocyte ratio; LMR, lymphocyte-to-monocyte ratio; AISI, aggregate index of systemic inflammation; SII, systemic immune-inflammation index; SIRI, systemic inflammatory response index. Panel descriptions: (**A**): PLR did not differ significantly between the groups (*p* > 0.05). (**B**): LMR was significantly lower in the unfavorable outcome group (*p* < 0.001). (**C**): NLR was significantly higher in patients with unfavorable outcomes (*p* < 0.001). (**D**): AISI was significantly higher in the unfavorable outcome group (*p* < 0.001). (**E**): SII was significantly higher in the unfavorable outcome group (*p* < 0.001). (**F**): SIRI was significantly higher in the unfavorable outcome group (*p* < 0.001).

The distribution of log-transformed inflammatory indices across the study groups was analyzed using violin plots (Figure 2). As shown in the graphs, NLR, SII, AISI, and SIRI exhibited significant differences in data distribution between the “0—favorable outcome stroke” and “1—unfavorable outcome stroke” groups. The LMR also demonstrated notable differences, but with an inverse trend, and was higher in the favorable outcome stroke group. In contrast, the PLR did not differ significantly between the groups.

In an exploratory stratified analysis by stroke subtype, inflammatory indices were compared separately in patients with ischemic and hemorrhagic stroke (Table 2). NLR, LMR, AISI, SIRI, and SII values were significantly higher in patients with unfavorable outcomes compared to those with favorable outcomes in both stroke subtypes (all *p* < 0.001). No statistically significant differences were observed between unfavorable ischemic and unfavorable hemorrhagic stroke groups, suggesting that the prognostic value of these indices reflects overall disease severity rather than stroke subtype. Based on our analyses, SIRI and NLR showed the strongest associations with unfavorable outcomes, demonstrating the highest odds ratios and AUC values among the studied indices. These findings are consistent with growing evidence that systemic inflammation is closely linked to stroke severity and early neurological deterioration.

## 4. Discussion

In the present study, age was independently associated with an increased risk of unfavorable outcome. This association remained statistically significant in the multivariable logistic regression analysis after adjustment for sex, stroke type, and inflammatory indices. This indicates that age contributes to stroke outcome independently of systemic inflammatory markers. This finding is consistent with previous studies showing that age is an important factor influencing stroke prognosis, regardless of stroke subtype [18]. The association between age and outcome may reflect differences in baseline physiological resilience and recovery capacity, rather than differences in inflammatory activity alone.

Among the inflammatory indices analyzed, SIRI and NLR showed the strongest associations of unfavorable outcomes. These indices demonstrated the highest odds ratios and AUC values among all markers analyzed. This finding supports growing evidence that systemic inflammation is closely associated with stroke severity and early neurological deterioration.

The association between higher SIRI values and unfavorable outcomes may be explained by the combined involvement of neutrophils, lymphocytes, and monocytes in post-stroke inflammatory and immune responses. Elevated neutrophil counts reflect activation of inflammatory pathways and oxidative stress, which may contribute to secondary brain injury [19]. At the same time, lymphopenia reflects dysregulation of the post-stroke immune response and is associated with increased vulnerability to complications. Increased monocyte counts indicate activation of systemic inflammatory mechanisms. Together, these changes suggest an imbalance between inflammatory activation and immune suppression after stroke. Such immune processes may contribute to a more severe disease course and higher risk of in-hospital mortality [20]. Dang et al. reported that an elevated SIRI (≥4.57) predicted higher 30-day, 90-day, and 1-year mortality and was an independent risk factor for poor prognosis in ischemic stroke [21]. Zhou et al. showed that a one-unit increase in SIRI was associated with a nearly 59% increase in the risk of unfavorable outcomes [22]. Combined with the ROC analysis, these findings suggest that SIRI may serve as a useful and accessible marker associated with adverse outcomes after stroke.

The prognostic value of NLR has also been widely reported. NLR reflects the balance between neutrophil-mediated inflammatory activation and lymphocyte-related immune regulation. Previous studies have shown that elevated NLR is associated with increased mortality and complications after ischemic stroke [23,24,25]. The results of the present study further support the role of NLR as a simple and practical indicator of adverse outcomes in acute stroke.

Because AISI combines several hematological parameters, it reflects the overall intensity of systemic inflammation. In this study, higher AISI values were associated with unfavorable outcomes. Similar findings have been reported in previous studies, which showed that elevated AISI values were related to increased mortality in both ischemic and hemorrhagic stroke [26,27,28].

Taken together, SIRI, NLR, SII, and AISI reflect different components of the inflammatory response, including neutrophil activation, lymphocyte suppression, and thromboinflammatory processes. Elevated values of these indices in patients with unfavorable outcomes likely reflect a more pronounced systemic inflammatory response and immune imbalance during the acute phase of stroke [29,30].

In this study, no significant differences were observed in the prognostic value of inflammatory indices between ischemic and hemorrhagic stroke. This finding suggests that these markers primarily reflect overall disease severity rather than stroke subtype-specific mechanisms. However, ischemic and hemorrhagic strokes differ in their underlying pathophysiology, and the absence of subtype-specific differences should be interpreted cautiously. The lack of statistically significant differences may be related to limited statistical power in subgroup analyses. Although stroke subtype was included as a covariate, larger studies are required to confirm these findings.

Further prospective, multicenter studies with standardized outcome measures are needed to confirm these findings and to clarify the clinical role of inflammatory hematological indices in stroke prognosis. It should be acknowledged that elevated inflammatory indices may reflect stroke severity, acute stress response, or concomitant infections rather than having a direct causal role in unfavorable outcomes. To reduce potential confounding, patients with acute infections and other active inflammatory conditions were excluded at baseline. Extreme values were removed using *Z*-score analysis, and blood samples were collected on the third day after hospitalization to minimize the influence of early stress-related hematological fluctuations. However, due to the retrospective design, residual confounding and reverse causation cannot be completely excluded. Therefore, the observed associations should be interpreted as prognostic relationships rather than evidence of direct causal effects.

### Limitations

This study had several limitations. Its retrospective design limited adjustment for all potential confounders. The sample size was relatively small and included patients from only two centers within a single region. Standardized neuroimaging data and detailed information on comorbidities, stroke severity, treatments, and in-hospital complications were limited. Concomitant infections and corticosteroid use were not fully accounted for, which may have influenced inflammatory marker levels. Standardized stroke severity measures, such as the National Institutes of Health Stroke Scale (NIHSS) based on imaging, were not consistently available in this retrospective dataset. In this study, the primary outcome was in-hospital mortality (survival versus death). Stroke severity is a major determinant of early mortality and is also likely to be associated with systemic inflammatory response. Therefore, the lack of adjustment for severity may limit the interpretation of the independent prognostic value of inflammatory indices. Elevated hematologic markers may partly reflect more severe initial neurological damage rather than acting as completely independent predictors of death.

An additional limitation is the exclusion of patients who died within the first 1–2 days of hospitalization. Because inflammatory indices were calculated using blood samples obtained on day 3 to minimize the influence of the hyperacute stress response, patients who did not survive until this time point were not included in the analysis. This may introduce survivor bias and limits the applicability of the findings primarily to patients who survived beyond the hyperacute phase of stroke.

## 5. Conclusions

In this retrospective study, SIRI and NLR showed the strongest associations with unfavorable outcomes in patients with both ischemic and hemorrhagic stroke. Their prognostic performance was similar across stroke types. The AISI index was also associated with overall inflammatory activity. These indices likely reflect the intensity of systemic inflammation during the acute phase of stroke. This information may be useful in clinical settings where access to advanced neuroimaging is limited. However, the inflammatory indices evaluated in this study should be interpreted as associative prognostic markers rather than validated clinical decision-making tools. Although they are derived from routine blood tests and are easy to obtain, their clinical utility requires further confirmation. Future prospective, multicenter studies with detailed clinical data and standardized neuroimaging are needed to validate these findings.

## Figures and Tables

**Table 1:** Comparison of inflammatory and hematological indices among study groups.

Index	Favorable Outcome	Unfavorable Outcome IS (Median, IQR)	*p*_1_ (Unfavorable IS vs. Favorable Outcome Stroke)	*r*_1_	Unfavorable HS (Median, IQR)	*p*_2_ (Unfavorable HS vs. Favorable Outcome Stroke)	*r*_2_	*p*_3_ (Unfavorable IS vs. Unfavorable HS)	*r*_3_
NLR	0.944 (0.560–1.29)	2.25 (1.48–2.68)	<0.001	0.723	2.02 (1.38–2.63)	<0.001	0.671	>0.05	0.0864
PLR	5.04 (4.77–5.21)	5.17 (4.78–5.52)	>0.05	0.188	5.05 (4.72–5.47)	>0.05	0.099	>0.05	0.0764
LMR	1.32 (1.01–1.61)	0.824 (0.351–1.30)	<0.001	0.426	0.822 (0.351–1.39)	<0.001	0.361	>0.05	0.0506
AISI	5.80 (5.37–6.11)	7.03 (6.29–7.72)	<0.001	0.657	7.01 (6.29–7.71)	<0.001	0.613	>0.05	0.0344
SIRI	0.91 (0.139–0.577)	1.69 (0.97–2.21)	<0.001	0.720	1.62 (0.72–2.29)	<0.001	0.667	>0.05	0.0446
SII	6.50 (6.25–6.87)	7.65 (6.86–8.19)	<0.001	0.668	7.42 (6.75–8.19)	<0.001	0.609	>0.05	0.0716

Values are presented as median (IQR) of ln-transformed variables. p_1_—comparison between the unfavorable ischemic stroke group and the favorable outcome group. p_2_—comparison between the unfavorable hemorrhagic stroke group and the favorable outcome group. p_3_—comparison between the unfavorable ischemic stroke group and the unfavorable hemorrhagic stroke group. r_1_, r_2_, and r_3_ represent the corresponding effect sizes for p_1_, p_2_, and p_3_, estimated using the Mann–Whitney U test. All *p*-values were obtained using the Mann–Whitney U test. Abbreviations: IS, ischemic stroke; HS, hemorrhagic stroke; IQR, interquartile range; NLR, neutrophil-to-lymphocyte ratio; PLR, platelet-to-lymphocyte ratio; LMR, lymphocyte-to-monocyte ratio; AISI, aggregate index of systemic inflammation; SIRI, systemic inflammatory response index; SII, systemic immune-inflammation index.

**Table 2:** Predictive performance of inflammatory and hematological indices for unfavorable stroke outcomes.

Index	AUC (95% CI)	*p*-Value	Cut-Off (Log)	Cut-Off (Original)	Sensitivity %	Specificity %	OR (95% CI)
NLR	0.885 (0.80–0.90)	<0.001	1.78	4.8	70.0	94.2	1.647 (1.37–1.97)
PLR	0.577 (0.49–0.66)	<0.008	5.43	199.4	41.5	84.1	1.004 (1.001–1.010)
LMR	0.702 (0.63–0.77)	<0.004	1.92	1.9	43.1	95.6	0.806 (0.7–0.93)
AISI	0.825 (0.76–0.88)	<0.001	6.67	530.6	76.1	85.5	1.003 (1.002–1.004)
SIRI	0.867 (0.79–0.90)	<0.001	1.33	2.7	71.5	97.1	2.360 (1.7–3.2)
SII	0.851 (0.77–0.88)	<0.001	7.14	1251.2	69.2	94.2	1.002 (1.001–1.003)

Cut-off (log) indicates the optimal threshold derived from ln-transformed values. Cut-off (original) indicates the corresponding threshold in the original (non-transformed) scale. Sensitivity and specificity are presented as percentages. *p*-values were obtained from ROC curve analysis. Odds ratios (ORs) with 95% confidence intervals (CIs) were calculated using logistic regression models with continuous predictors. Abbreviations: ROC, receiver operating characteristic curve; AUC, area under the curve; CI, confidence interval; OR, odds ratio; NLR, neutrophil-to-lymphocyte ratio; PLR, platelet-to-lymphocyte ratio; LMR, lymphocyte-to-monocyte ratio; AISI, aggregate index of systemic inflammation; SIRI, systemic inflammatory response index; SII, systemic immune-inflammation index.

**Table 3:** Multivariable logistic regression analysis of factors independently associated with unfavorable in-hospital outcome.

Variable	Odds Ratio (OR)	95% CI	*p*-Value
Age (per 1-year increase)	1.09	1.03–1.15	0.003
NLR	1.71	1.25–2.34	<0.001
SIRI	1.56	0.96–2.52	0.073
Stroke type (hemorrhagic vs. ischemic)	17.9	4.69–68.34	<0.001
Sex (female vs. male)	0.74	0.29–1.91	0.531

Odds ratios (OR) and 95% confidence intervals (CI) were obtained from multivariable logistic regression analysis. In-hospital mortality was used as the dependent variable (0 = survived, 1 = died). Age was analyzed as a continuous variable (per 1-year). Sex and stroke type were entered as binary variables. NLR and SIRI were included as continuous predictors. Abbreviations: OR, odds ratio; CI, confidence interval; NLR, neutrophil-to-lymphocyte ratio; SIRI, systemic inflammatory response index.

## Data Availability

The datasets generated and analyzed during this study are available upon request from the corresponding author.

## Abbreviations

NLR	neutrophil-to-lymphocyte ratio
PLR	platelet-to-lymphocyte ratio
LMR	lymphocyte-to-monocyte ratio
SIRI	systemic inflammatory response index
SII	systemic immune-inflammation index
AISI	aggregate index of systemic inflammation
IS	ischemic stroke
HS	hemorrhagic stroke
ROC	receiver operating characteristic
AUC	area under the curve
